# Deep learning models for histologic grading of breast cancer and association with disease prognosis

**DOI:** 10.1038/s41523-022-00478-y

**Published:** 2022-10-04

**Authors:** Ronnachai Jaroensri, Ellery Wulczyn, Narayan Hegde, Trissia Brown, Isabelle Flament-Auvigne, Fraser Tan, Yuannan Cai, Kunal Nagpal, Emad A. Rakha, David J. Dabbs, Niels Olson, James H. Wren, Elaine E. Thompson, Erik Seetao, Carrie Robinson, Melissa Miao, Fabien Beckers, Greg S. Corrado, Lily H. Peng, Craig H. Mermel, Yun Liu, David F. Steiner, Po-Hsuan Cameron Chen

**Affiliations:** 1 Google Health, Palo Alto, CA USA; 2 Work done at Google Health via Vituity, Emeryville, CA USA; 3 Work done at Google Health, current affiliation Tempus Labs Inc, Chicago, IL USA; 4 Department of Pathology, School of Medicine, University of Nottingham, Nottingham, UK; 5 John A. Burns University of Hawaii Cancer Center, Honolulu, HI USA; 6 Department of Pathology, Magee-Womens Hospital of UPMC, Pittsburgh, PA USA; 7 Defense Innovation Unit, Mountain View, CA USA; 8 Uniformed Services University, Bethesda, MD USA; 9 Henry M. Jackson Foundation, Bethesda, MD USA; 10 Laboratory Department, Naval Medical Center San Diego, San Diego, CA USA; 11 Verily Life Sciences, South San Francisco, CA USA

**Keywords:** Breast cancer, Breast cancer, Cancer imaging

## Abstract

Histologic grading of breast cancer involves review and scoring of three well-established morphologic features: mitotic count, nuclear pleomorphism, and tubule formation. Taken together, these features form the basis of the Nottingham Grading System which is used to inform breast cancer characterization and prognosis. In this study, we develop deep learning models to perform histologic scoring of all three components using digitized hematoxylin and eosin-stained slides containing invasive breast carcinoma. We first evaluate model performance using pathologist-based reference standards for each component. To complement this typical approach to evaluation, we further evaluate the deep learning models via prognostic analyses. The individual component models perform at or above published benchmarks for algorithm-based grading approaches, achieving high concordance rates with pathologist grading. Further, prognostic performance using deep learning-based grading is on par with that of pathologists performing review of matched slides. By providing scores for each component feature, the deep-learning based approach also provides the potential to identify the grading components contributing most to prognostic value. This may enable optimized prognostic models, opportunities to improve access to consistent grading, and approaches to better understand the links between histologic features and clinical outcomes in breast cancer.

## Introduction

Breast cancer is the most common cancer in women and one of the leading causes of cancer death worldwide^1^. The heterogeneous nature of breast cancer makes its initial characterization a critical step in treatment planning and decision making. One aspect of breast cancer characterization that remains central to its prognostic classification is the Nottingham combined histologic grade (Elston-Ellis modification of Scarff-Bloom-Richardson grading system)^2,3^. First described and validated over 30 years ago^4^, the Nottingham Grading System (NGS) consists of three components: mitotic count (MC), nuclear pleomorphism (NP), and tubule formation (TF), and is an important component of existing prognostic tools including the AJCC prognostic stage grouping^5^, PREDICT online prognostic classification tool^6^, and the Nottingham Prognostic Index^7^. However, while the combined histologic grade has been repeatedly shown to be associated with clinical outcomes, the task’s inherent subjectivity can also result in inter-pathologist variability that limits the generalizability of its prognostic utility^2,8^. In addition, up to half of breast cancer cases are classified in routine practice as “grade 2”, an intermediate risk group with limited clinical value due to inclusion of some low and high-grade tumors^3^.

The application of computer vision and artificial intelligence (AI) to histopathology has seen tremendous growth in recent years and offers the potential to augment pathologist expertise and increase consistency and efficiency. Work relevant to breast cancer includes AI systems for counting mitoses^9–12^, scoring nuclear pleomorphism^13,14^, recognizing tumor subtypes^15,16^, detecting metastases in lymph nodes^17,18^, identifying biomarker status^19–23^, and predicting prognosis^24–26^. While such prior works address automated breast cancer grading, they have not specifically combined models for all three components of the Nottingham grading system and only a small number of used prognostic evaluation to complement the validation approach. Additional differences to consider across works include the machine learning approach and the image and specimen type, such as direct microscope image capture^15^, tissue microarray^14^, or whole slide images. Understanding the performance and application of such tools in the context of actual pathological review and workflows remains a critical next step for translation to clinical utility.

Uniquely, this work represents the development and combination of deep learning models for all three components of the NGS, with evaluation against reference grades provided by expert review of multiple pathologists. To further complement evaluation against pathologist grading and to explore the use of these automated tumor grading models, we analyze the prognostic value of the AI-based tumor grades. Prognostic evaluation utilizes an external test set consisting of cases from the The Cancer Genome Atlas breast invasive carcinoma (TCGA BRCA) study. This analysis demonstrates prognostic value on par with that of tumor grading provided by pathologists, providing additional validation of the AI-based Nottingham grading system (AI-NGS) and providing a potential approach to improve breast cancer classification and prognostication. By enabling grading that is both more objective (less inter-pathologist variability) and more fine grained (via availability of continuous scores for each component), the AI-NGS can combine strengths of AI with existing knowledge about the prognostic value of well-established morphological features^14^.

## Results

### Cohort characteristics

All available whole-slide images (WSIs) from three data sources were reviewed by qualified pathologists for slide-level inclusion criteria and quality assurance (see Methods). This resulted in 657 cases (1502 slides) from a tertiary teaching hospital (TTH), 98 cases (98 slides) from a medical laboratory (MLAB), and 829 cases (878 slides) from TCGA. TTH and MLAB were used for model development, while TCGA was used for evaluation only. The datasets and corresponding clinical characteristics are summarized in Table 1. For the test set, 829 TCGA cases (878 slides) were used for prognostic evaluation and 662 TCGA cases (685 slides) with available reference annotations were used for evaluation of histologic grading (case inclusion and exclusion are summarized in Supplementary Fig. 1).

**Table 1:** Dataset characteristics for development and evaluation of grading and outcome prediction models.

A				
Data Usage	Train	Tune		Test
DLS Stage 1 (patch-level)				
Data source	TTH	TTH		TCGA
No. of cases	405	142		662
No. of H&E slides	520	148		685
DLS Stage 2 (slide-level)				
Data source	TTH	TTH	MLAB	TCGA
No. of cases	261	212	98	662
No. of H&E slides	551	431	98	685
Case-level ER* (pos / neg)	144/33	92/38	89/7	468/147
Slide-level MC score (1/2/3)	368/47/81	214/59/106	78/10/7	193/120/145
Slide-level NP score (1/2/3)	58/294/146	19/180/189	21/57/16	31/235/237
Slide-level TF score (1/2/3)	55/87/357	37/47/303	13/18/64	9/93/369

B		
Data usage	Tune	Test
Prognostic Models (Cox Regression)		
Data source	TTH	TCGA
No. of cases	354	829
No. of H&E slides	829	878
Age (min, max, median, mean, std)	25/97/58/57.8/13.2	26/90/58/58.4/13.0
Follow-up min/max/median/mean/std (days)	398/5830/2692/2816/1412	0/8556/788/1200/1115
No. of events (PFI)	60	93
No. of censored events at 5-year	246	166
Tumor size		
T1	199	221
T2	112	476
T3	22	107
T4	5	23
Lymph node		
N0	203	404
N1	65	261
N2+	31	148
Metastasis		
M0 (negative)	1	808
M1 (positive)	NA	13
Hormone receptor status		
ER* (pos / neg)	275/73	593/177
Nottingham Grading System		
MC score (1/2/3)	213/42/94	247/147/172
NP score (1/2/3)	16/206/127	38/299/286
TF score (1/2/3)	34/61/354	14/127/444

### Performance of deep learning systems for component features

**Fig. 1:**
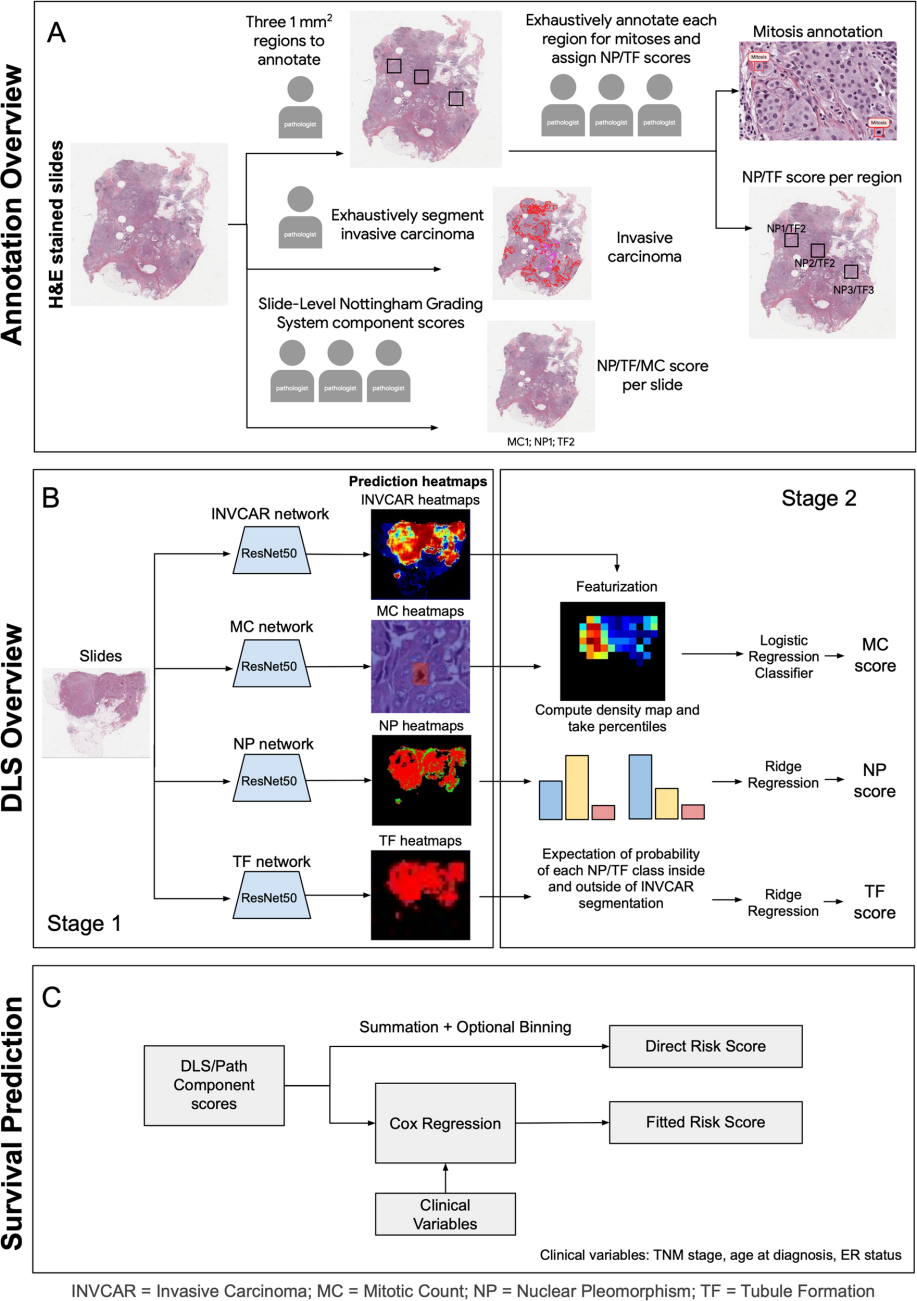
Overview of annotation, deep learning system (DLS) development, and prognostic evaluation. **A** Annotation Overview: Pathologists provided annotations at a region-level and slide-level for all components of the histologic grade, including identification of individual mitotic figures. **B** DLS overview: convolutional neural network models were developed for invasive carcinoma (INVCAR) as well as all three components of the histologic grading system. These patch-level models were used as input to stage 2 models to provide a component score at the slide level for each feature. **C** Prognostic evaluation: Component grade scores provided by the DLS or pathologists were used to fit Cox models for evaluation and comparison of prognostic value.

**Fig. 2:**
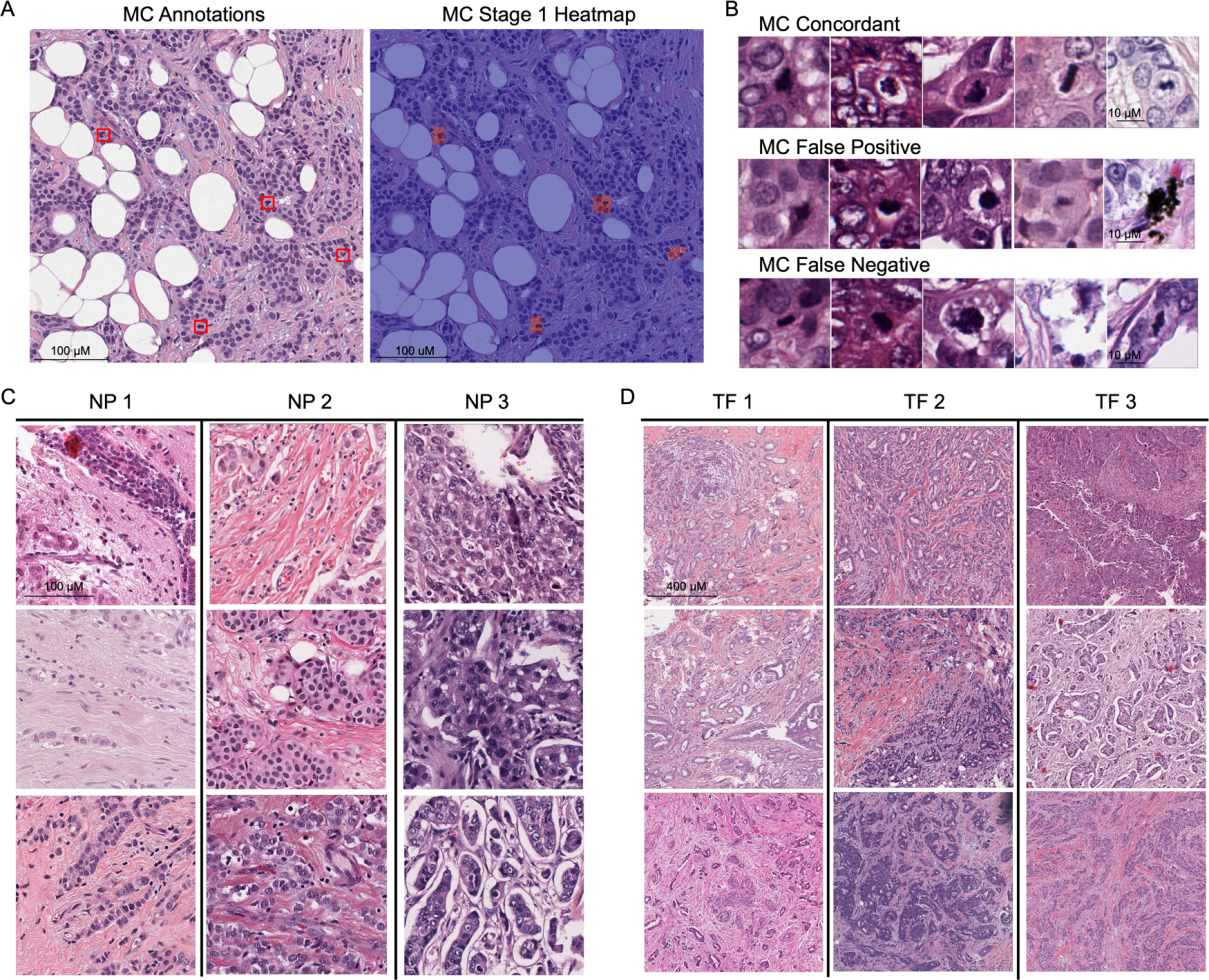
Visualization of patch-level DLS predictions. **A** Pathologist annotations for mitoses corresponding to a single high-power field of 500 μm × 500 μm (left) and the corresponding heatmap overlay provided by the MC model (right). Red regions of the overlay indicate high likelihood of a mitotic figure according to the model. **B** Patches corresponding to regions for which pathologists and the model both identified mitotic figures (concordant), regions classified as mitotic figures by the model but not identified by at least 2 of 3 reviewing pathologists (false positive), or regions identified by at least 2 of 3 reviewing pathologists as containing a mitotic figure but not classified as such by the model. Discordant identification of mitotic figures by the model did not appear to be due to image or staining quality, but rather, appeared to largely reflect morphologic details for which pathologist identification of mitotic figures was also variable. Patches are 32 μm x 32 μm with scale bar of 10 μm shown in the top row. **C** Individual patches classified as grade 1, 2, 3 for nuclear pleomorphism. (40× magnification; 256 μm × 256 μm; 100 μm scale bar for reference). **D** Individual patches classified as grade 1, 2, or 3 for tubule formation (10× magnification; 1 mm × 1 mm; 400 μm scale bar for reference). MC mitotic count, NP nuclear pleomorphism, TF tubule formation.

We developed individual deep learning systems (DLS) for each component of the Nottingham grading system (MC, NP, TF). The scores generated by the DLS for each feature are continuous, and can then be discretized to produce integer scores (1,2,3) for comparison to pathologist grading. We evaluated the performance of each DLS on the held-out test set of WSIs from TCGA using majority vote reference annotations provided by three pathologists (approach summarized in the schematic in Fig. 1). The individual component models were evaluated at both the “patch-level” and the “slide-level” (see Methods). Patch-level performance results are summarized in Table 2 with example classifications from the individual models shown in Fig. 2. For the mitosis detection model (evaluated as a detection task), the mitotic figure F1 score was 0.60 (95% CI: 0.58, 0.62). For the patch-level classification models, the quadratic Kappa was 0.45 (95% CI: 0.41, 0.50) for NP and 0.70 (95% CI: 0.63, 0.75) for TF. Examples of patch-level predictions across entire WSIs are provided in Supplementary Fig. 2. For evaluation at the slide-level, using the majority score provided by pathologists as the reference grade, quadratic-weighted kappa was 0.81 (95% CI: 0.78, 0.84) for MC, 0.48 (95% CI: 0.43, 0.53) for NP, and 0.75 (95% CI: 0.67, 0.81) for TF (Table 2). Additional metrics to enable comparison to other studies (including unweighted kappa and precision and recall for MC) are available in Supplementary Table 1 and benchmark comparisons for slide-level grading by both pathologists and computational approaches are provided in Supplementary Table 2.

**Table 2:** Component model performance.

	Nottingham Components	Metric	Result [95% CI]
Patch-level	Mitotic Count	F1-Score	0.60 [0.58, 0.62]
	Nuclear Pleomorphism	Quadratic-weighted Kappa	0.45 [0.41, 0.50]
	Tubule Formation	Quadratic-weighted Kappa	0.70 [0.63, 0.75]
Slide-level	Mitotic Count	Quadratic-weighted Kappa	0.81 [0.78, 0.84]
	Nuclear Pleomorphism	Quadratic-weighted Kappa	0.48 [0.43, 0.53]
	Tubule Formation	Quadratic-weighted Kappa	0.75 [0.67, 0.81]

**Fig. 3:**
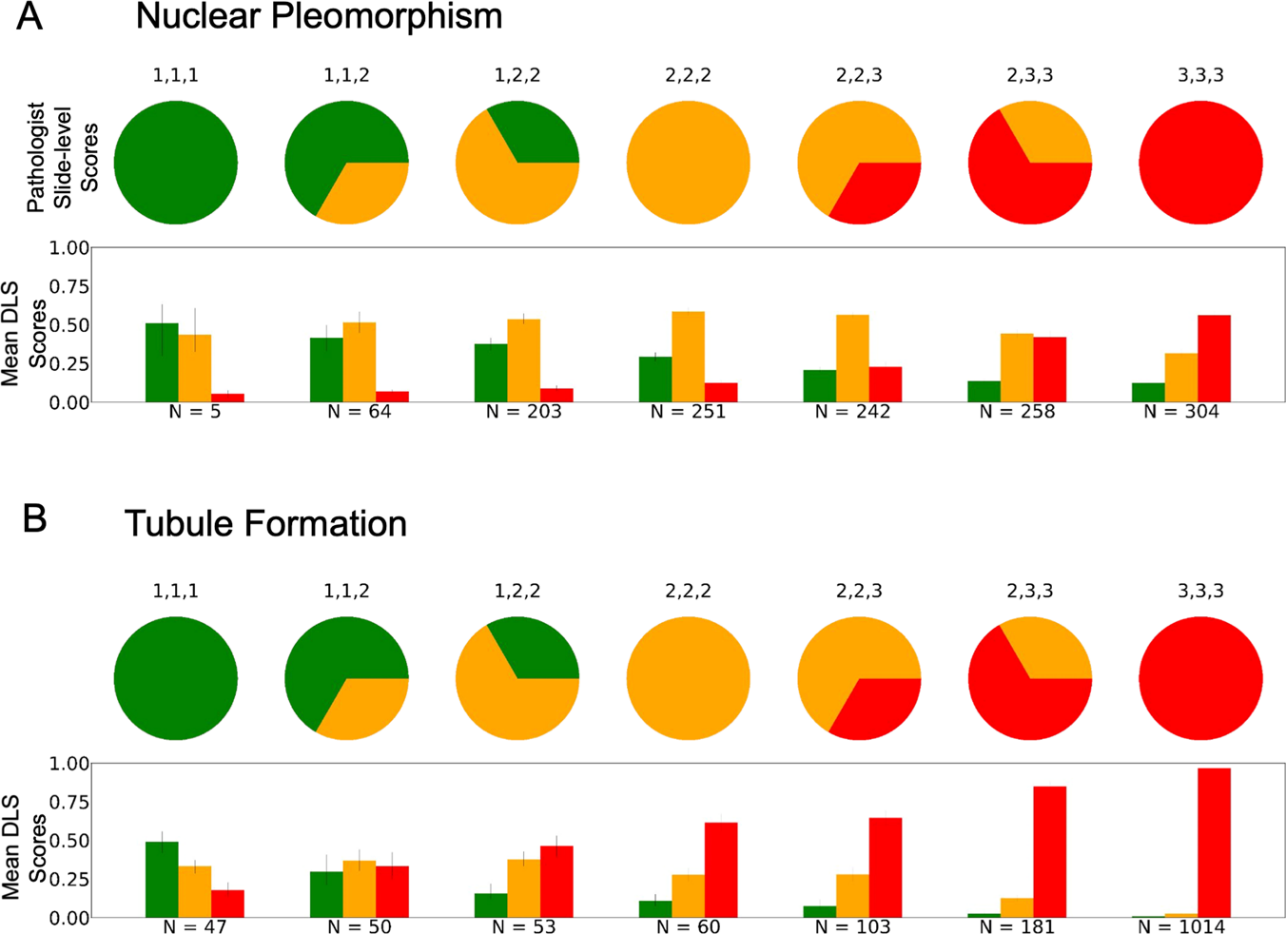
Assessing slide-level classification of nuclear pleomorphism and tubule formation. The three pathologist scores provided for nuclear pleomorphism (**A**) and Tubule Formation (**B**) for each slide are represented by the pie charts. Bar plots represent the model output for each possible component score with the mean output plotted for the cases matched to those represented by the corresponding pie chart. Green corresponds to component score of 1, yellow to component score of 2, and red to component score of 3. Error bars are 95% CI. DLS Deep Learning System.

One challenge in the performance evaluation of deep learning models for histologic grading is that pathologist-provided grading itself can be subject to high inter-rater variability^27^. Given the availability of three pathologist reviews for each slide in our study (see Annotations section of Methods), we grouped slides by the combination of pathologist scores for each slide and evaluated the DLS output for the resulting groups (Fig. 3). This analysis demonstrates that the continuous nature of the DLS output can reflect the distribution of pathologist agreement, whereby the output of the deep learning models produces “intermediate scores” for cases lacking unanimous pathologist agreement. For example, a case with a majority vote score of “1” for nuclear pleomorphism may have unanimous agreement across all three pathologists, or may have one pathologist giving a higher score, and the models were found to reflect these differences. As seen in Fig. 3, as fewer pathologists indicated a score of “1” and more pathologists indicated a score of “2” or “3”, the DLS-estimated probability for a score of “1” (in green) decreased, and the estimated probability for a score of “3” (in red) increased.

**Fig. 4:**
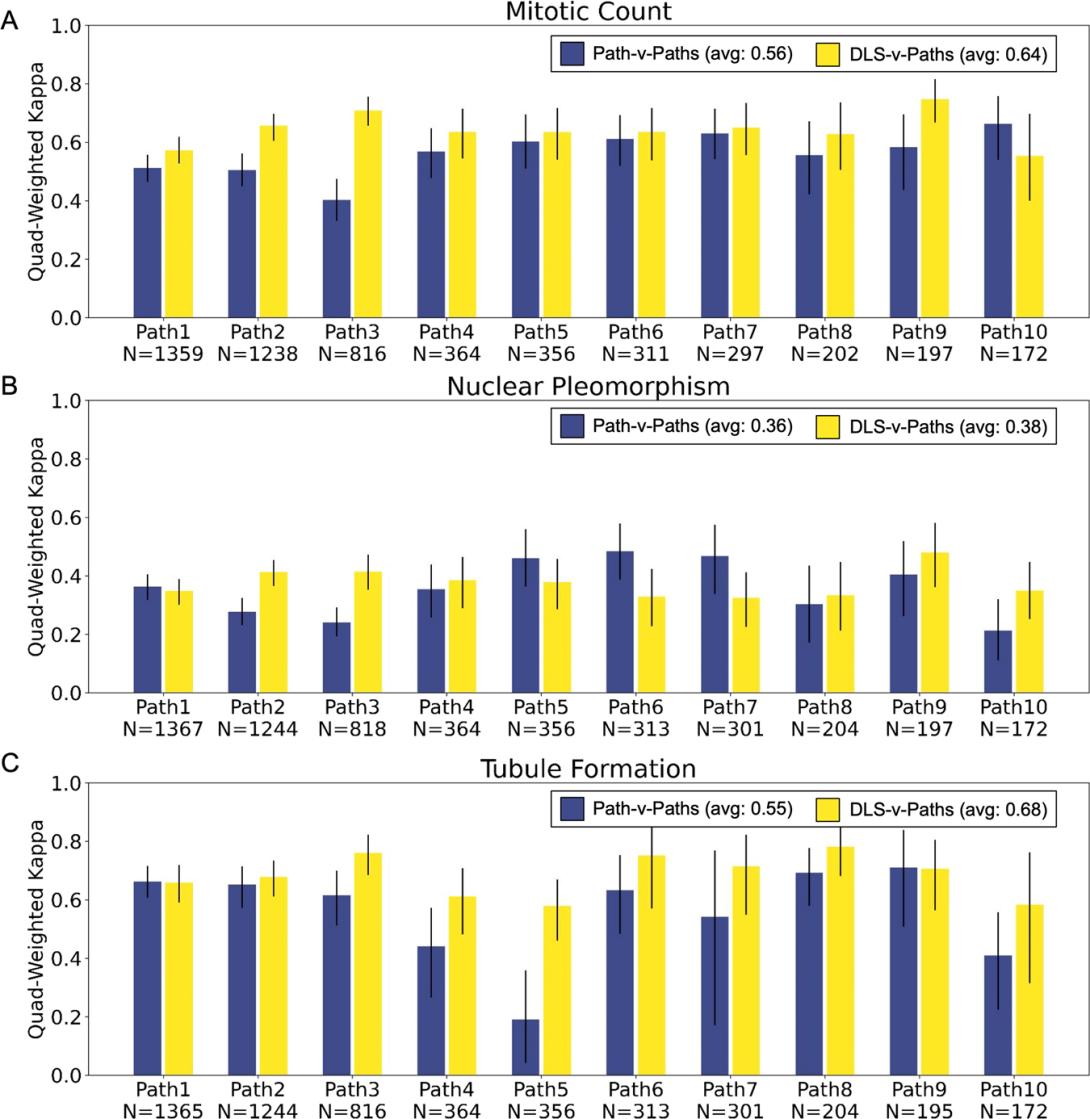
Inter-pathologist and DLS-pathologist concordance for slide-level component scoring. Analyses for mitotic count (**A**), nuclear pleomorphism (**B**), and tubule formation (**C**) are shown in the individual panels. Each blue bar represents the agreement (quadratic-weighted kappa) between a single pathologist and the other available pathologist scores on the same cases. The yellow bar represents the agreement of the DLS-provided component score with all available pathologist’ scores on the matched set of cases. Error bars represent 95% confidence intervals computed via bootstrap. Average values in legend represent quadratic-weighted kappa or the average of all blue bars and yellow bars, respectively. DLS deep learning system, Paths pathologists.

Next, to further evaluate DLS performance in the context of known inter-rater variability we calculated both inter-pathologist and DLS-pathologist agreement. The average kappa (quadratic-weighted) for inter-pathologist agreement was 0.56, 0.36, 0.55 for MC, NP, and TF respectively, versus 0.64, 0.38, 0.68 for DLS-Pathologist agreement (Fig. 4). The kappa for inter-pathologist agreement for each individual pathologist (one vs. rest) as well for DLS-pathologist agreement are summarized in Fig. 4, demonstrating that on average the DLS provides consistent, pathologist-level agreement on grading of all three component features. The full confusion matrices for inter-pathologist agreement and for DLS agreement with the majority vote scores (patch-level and slide-level) are available in Supplementary Figs. 3 and 4.

### Prognostic value of AI-NGS

We further analyzed the association of both AI-NGS and pathologist-provided grades with clinical outcomes, using the external test dataset (TCGA-BRCA) and progression free interval (PFI) as the prognostic endpoint^28^. We conducted non-inferiority analysis comparing AI-NGS to histologic grading provided by pathologists. Based on tune set results, the planned, primary comparison for this analysis used the sum of the continuous component scores generated by AI-NGS (AI-NGS continuous sum; float value 3–9) compared to the summed discrete score provided by pathologist review (pathologist discrete sum; integer value 3–9); see PFI Analysis section of the Methods for additional details of continuous versus discrete scoring). The prognostic performance of the two approaches were similar with a c-index of 0.58 (95% CI: 0.52, 0.64) using the AI-NGS continuous sum and 0.58 (95% CI: 0.51, 0.63) using the pathologist discrete sum (Table 3; delta = 0.004, lower bound of one-sided 95% CI: −0.036). This is consistent with non-inferiority of AI-NGS relative to pathologist grading (see Statistical Analysis section of Methods for additional details).

**Table 3:** Prognostic performance of direct risk prediction using histologic scoring provided by DLS and pathologists.

Scoring method	C-index (All cases; *n* = 829)	
	DLS	Pathologist
Combined Histologic Grade [1,2,3]	0.60 [0.55, 0.65]	0.58 [0.51, 0.63]
Summed Score Discrete [3,4,5,6,7,8,9]	0.59* [0.53, 0.64]	0.58* [0.51, 0.63]
Summed Score Continuous [3.00–9.00]	0.58* [0.52, 0.64]	N/A

*DLS* deep learning system.

*Planned non-inferiority test comparing these configurations: DLS Summed Score Continous and Pathologist Summed Score Discrete.

While the continuous scores of the AI-NGS were utilized for primary analysis based on superior performance of this approach on the tune set (Supplementary Table 3) as well as prior work in prostate cancer^29^, additional approaches were also evaluated, including use of discrete summed scores (values 3–9) and the combined histologic grade (grade 1–3 based on the summed score^4^). For pathologist grading, majority vote and originally reported diagnostic grading were also evaluated. Performance for these various scoring configurations summarized in Supplementary Table 4. The c-index for AI-NGS approaches were similar, 0.58 (95% CI: 0.52, 0.64) for the AI-NGS continuous sum, 0.59 (95% CI: 0.53, 0.64) for the AI-NGS discrete sum, and 0.60 (95% CI: 0.55, 0.65) for the combined histologic grade. The pathologist-based approaches were also similar, ranging from 0.58 (95% CI: 0.51, 0.63) for pathologist combined histologic grades (1–3) to 0.61 (95% CI: 0.54, 0.66) for the majority vote summed score (3–9). The association of each individual grading component with prognosis was also evaluated independently (Supplementary Table 5). The highest prognostic value for deep learning-based grading of a single feature on the test set was achieved for mitotic count, with a c-index of 0.58 (95% CI: 0.53, 0.64). The pathologist’s mitotic count score gave a c-index of 0.54 (95% CI: 0.48, 0.59).

We also evaluated the prognostic value of AI-NGS in the context of established baseline variables (ER status, tumor size, nodal involvement, and age). Overall, adding AI-NGS provided improved prognostic value over the baseline variables alone (*p* = 0.036; likelihood ratio test of full model versus baseline model; Table 4). To better understand the potential contribution of each component feature, we performed a similar analysis using the score for each feature independently. Analysis for each component feature individually suggested improved prognostic value specifically for the AI-based mitotic count score (*p* = 0.041; likelihood ratio test) but not the other features (Supplementary Table 6). Additionally, in univariable hazard ratio (HR) analysis, the MC score provided the only AI-NGS feature with a *p* value less than 0.05 (HR = 1.30, *p* = 0.015; univariable HR analysis) (Table 5). In multivariable analysis adjusting for ER status, tumor size (T-category), nodal involvement, and age this corresponded to a HR of 1.29 (*p* = 0.061; multivariable HR analysis) (Table 5).

**Table 4:** Prognostic performance using summation of histologic components in combination with baseline clinical and pathologic features.

Model features	c-index	*p*-value (likelihood ratio test) for adding features to baseline
Baseline Features Only	0.74 [0.67, 0.81]	N/A (reference)
Baseline + AI-NGS	0.76 [0.69, 0.81]	0.036
Baseline + Single Pathologist	0.75 [0.69, 0.81]	0.064
Baseline + Majority Pathologist	0.76 [0.70, 0.82]	0.023

Cox models were fitted and evaluated directly on the test set and p-values are for likelihood ratio test of baseline versus baseline plus grading scores. Baseline features include age (continuous), TNM (categorical), and ER status (binary). Number of cases represents all cases with baseline characteristics available (*n* = 762 cases; 82 events). Majority pathologist refers to the majority voted scores of three pathologists. Confidence intervals computed via bootstrap with 1000 iterations.

**Table 5:** Cox regression on the test set using pathologist grading or AI-NGS scores and baseline variables.

	Univariable analysis				Multivariable analysis			
Feature	Pathologist		AI-NGS		Pathologist		AI-NGS	
	HR	*p*	HR	*p*	HR	*p*	HR	*p*
Age	1.01 [0.99, 1.02]	0.378	1.01 [0.99, 1.02]	0.213	1.01 [0.99, 1.03]	0.230	1.01 [0.99, 1.03]	0.213
ER status								
Negative	1.0 (ref)	N/A	1.0 (ref)	N/A	1.0 (ref)	N/A	1.0 (ref)	N/A
Positive	0.59 [0.39, 0.91]	0.017	0.60 [0.39, 0.92]	0.018	0.54 [0.33, 0.86]	0.010	0.55 [0.33, 0.89]	0.016
Metastasis present								
Negative	1.0 (ref)	N/A	1.0 (ref)	N/A	1.0 (ref)	N/A	1.0 (ref)	N/A
Positive	14.03 [7.36, 26.76]	<0.001	14.09 [7.39, 26.88]	<0.001	11.54 [5.58, 23.87]	<0.001	10.31 [4.85, 21.93]	<0.001
Nodal status								
N0	1.0 (ref)	N/A	1.0 (ref)	N/A	1.0 (ref)	N/A	1.0 (ref)	N/A
N1+	2.09 [1.41, 3.09]	<0.001	2.10 [1.42, 3.11]	<0.001	1.65 [1.05, 2.57]	0.029	1.70 [1.09, 2.66]	0.020
Tumor Size								
T 1	1.0 (ref)	N/A	1.0 (ref)	N/A	1.0 (ref)	N/A	1.0 (ref)	N/A
T2	0.89 [0.61, 1.30]	0.559	0.90 [0.62, 1.30]	0.566	0.99 [0.60, 1.64]	0.981	0.96 [0.58, 1.60]	0.880
T3+	2.38 [1.56, 3.64]	<0.001	2.39 [1.56, 3.65]	<0.001	2.28 [1.30, 4.02]	0.004	2.36 [1.33, 4.18]	0.003
MC	1.27 [1.02, 1.56]	0.028	1.30 [1.05, 1.61]	0.015	1.01 [0.78, 1.29]	0.959	1.29 [0.99, 1.69]	0.061
NP	1.16 [0.86, 1.58]	0.336	0.97 [0.69, 1.37]	0.879	1.26 [0.87, 1.83]	0.226	0.91 [0.60, 1.37]	0.649
TF	1.60 [1.03, 2.50]	0.038	1.35 [0.89, 2.04]	0.157	1.79 [1.04, 3.07]	0.034	1.50 [0.92, 2.45]	0.102

For individual component scores (MC, NP, and TF), the discrete component scores were used (values are 1, 2, or 3 for each component).

### Mitotic count and Ki-67 expression

**Fig. 5:**
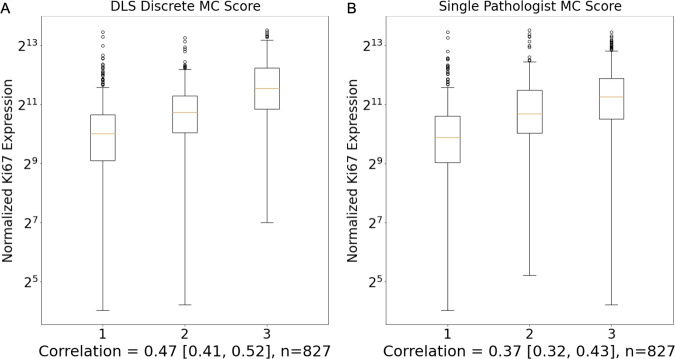
Correlation of mitotic count and Ki-67 expression. Correlation of mitotic count and Ki-67 gene expression is shown for the mitotic count provided by the DLS (**A**) and by pathologist review (**B**). Values in brackets below plots represent 95% confidence interval for correlation. Box plot box edges and center line indicate quartiles (25th, 50th, and 75th percentile) of Ki-67 gene expression (TPM values via TCGA) for each MC score, with outliers (defined as values beyond 1.5 times the interquartile range) plotted as black circles. The correlation values for DLS MC scoring with Ki-67 (0.47) and pathologist MC scoring with Ki-67 (0.37) were significantly different (*p* = 0.002; permutation test) based on a permutation test with *n* = 1000 samples. DLS deep learning system, MC Mitotic Count.

Given the potential association between MC, Ki-67, and prognosis, and the increasing interest of the clinical community in the use of Ki-67 in breast cancer^30,31^, we also evaluated the correlation between MC score and Ki-67 (*MKI67*) gene expression in our study (Fig. 5). The MC score provided by the DLS demonstrated correlation with *MKI67* expression with a correlation coefficient of 0.47 (95% CI: 0.41, 0.52) across the 827 TCGA cases with available gene expression data. For pathologist-provided MC scores over the same cases, the correlation coefficient was 0.37 (95% CI: 0.32, 0.43). This indicates an increased correlation with Ki-67 for the DLS-provided MC score as compared to MC provided by pathologist review (*p* = 0.002 in exploratory analysis; permutation test).

## Discussion

In this study we developed deep learning models for all three components of the Nottingham histologic grading system, to perform both patch-level and slide-level prediction of these histologic features. A key feature of this work is that we use survival analyses to further evaluate our AI-NGS models using the more objective endpoint represented by clinical outcome. The performance for each component model exceeds most published benchmarks, and the model’s prediction of clinical outcome is shown to be on-par to that of pathologist-based grading. Simultaneous development of all three models enables a consistent, end-to-end DLS for Nottingham histologic grading that can also provide transparency into the underlying component features. While prior work has shown promising results for individual features^9,11–13^ or direct prediction of final combined histologic grade^26^, this work is unique in combining multiple patch-level component models for the Nottingham grading system. This also enables analysis of prognostic models that can take advantage of the scores for individual features.

One important challenge to accurate and useful histologic grading is the inherent subjectivity and associated inter-pathologist variability. As such, an automated DLS for histologic grading can provide internal consistency and reliability for grading any given tumor. Such models thus have the potential to be iteratively tuned and updated with expert pathologist oversight to correct error modes and stay consistent with evolving guidelines. Additionally, this study found that DLS-pathologist agreement generally avoids the high discordance that is sometimes observed between individual pathologists while overall trends for agreement across the three features were consistent with prior reports (Supplementary Table 2). Consistent, automated tools for histologic grading may help reduce discordant interpretations and mitigate the resulting complications that impact clinical care and research studies evaluating interventions, other diagnostics, and the grading systems themselves.

The continuous, consistent, and precise component scores provided by this approach also enable exploration of the individual components contributing most to the prognostic value. In our analysis, the AI-NGS provided significantly increased prognostic value relative to the baseline variables alone. Of the individual component features, MC demonstrated the strongest, independent association with PFI in our analysis, a finding consistent with prior studies^32,33^. Building on this, the finding that mitotic count estimation by the DLS correlates with Ki-67 gene expression has implications for ongoing research regarding integration of Ki-67 in prognostic models in breast cancer^34,35^. Also, the stronger correlation of Ki-67 with DLS mitotic count than with pathologist mitotic count suggests that for discordant mitotic figure classifications between DLS and pathologist (such as those in Fig. 2B) the DLS might in fact be providing more accurate representation of the biological ground truth (i.e., cell proliferation) than the pathologist-provided reference annotations. Additional studies using immunohistochemistry-based ground truth for training and evaluation^36^, as well as future comparisons between automated mitotic count and automated Ki-67 immunohistochemistry quantitation may provide useful insights into these approaches. Overall, AI-NGS can be applied in future studies to large, multi-institutional datasets, minimizing complications of inter-pathologist variability and without requiring additional pathologist case review. This may in turn help refine existing regression models such as the Nottingham Prognostic Index^37^ or Magee equations^38^, by enabling further optimization of weights and providing consistent, precise, and automated scoring at scale.

Interestingly, in our test set, the summed continuous DLS score (floating point values in [3,9]) was not more prognostic than using a discrete, less granular, combined histologic grade (grade 1, 2, or 3). This is despite the continuous score being slightly superior on the smaller TTH “tune” data split, and is in contrast to our related work in prostate cancer where continuous, DLS-based Gleason scoring was superior to discrete grade group categories for outcome prediction^29^. This may be due in part to the relatively large confidence intervals associated with the small rate of events as well as domain shifts between development and test sets due to inter-institutional differences or variability in slide processing and quality, especially given the diversity of tissue source sites in TCGA. Additionally, most TCGA cases only contributed a single slide, which may not always be most representative of the tumor and associated histologic features.

Limitations of this study include the following: While the training slides used in this study represent two institutions and the test set comprises multiple sites contributing to TCGA, further evaluation of generalizability to diverse cohorts and across individual tumor subtypes is warranted. Additionally, although TCGA has useful attributes as a test set for this study (eg, diversity of pre-analytical variables and tissue sites), the follow-up time is limited with a median of ~2 years. As much of the evidence for the prognostic significance of histological grading is in the setting of longer follow-up time to provide more complete recurrence event information^2^, the relatively shorter follow-up time available for TCGA data may result in less precise estimates of prognostic value both for pathologists and AI-based grading). This is likely to have a predominant impact on analysis of ER + HER2− cases, for which progression events often happen later yet for which grading is expected to provide the best risk stratification. As such, the limited follow-up time may partially explain the relatively modest C-indices observed when considering the grading in isolation for this cohort. An additional limitation is that we were not able to control for possible confounding due to treatment differences, as this information was not available for most cases. Future work utilizing datasets with longer clinical follow-up, treatment data, and larger cohorts that enable analysis of individual tumor subtypes may allow improved prognostic evaluation and further demonstrate the method’s clinical significance. Larger, diverse datasets may also enable model development that directly predicts the progression-free survival from the tissue images, without using histologic grade as an intermediate prediction to estimate clinical outcomes. Such “weakly supervised” approaches could allow identification of new associations between survival and morphological features, potentially leading to iterative refinement of existing grading systems. Lastly, given the subjective nature of the pathologist grading used as the reference standard for model evaluation, adjudication sessions to achieve consensus scoring and mitotic figure identification may improve the quality of labels for training and evaluation, and this hypothesis could be tested in future work.

This study demonstrates the potential for deep learning approaches to provide comprehensive grading in breast cancer that is on par with pathologist review. The consistent and precise nature of these models allows for potentially improved integration into prognostic models as well as enabling opportunities to efficiently evaluate correlations between morphological and molecular features. Future work that combines AI-based grading of established histologic features with additional machine-learned features to generate improved prognostic models remains a compelling next step.

## Methods

### Data

This retrospective study utilized de-identified data from three sources: a TTH, a MLAB, and TCGA. Histopathology, clinical data, and Ki-67 expression data for TCGA were accessed via https://portal.gdc.cancer.gov. WSIs from TTH include original, archived hematoxylin and eosin (H&E)-stained slides and freshly cut and stained sections from archival blocks. WSIs from MLAB represent freshly cut and H&E stained sections from archival blocks. All WSIs used in this study were scanned at 0.25 μm/pixel (40×). The small number of TCGA images in the BRCA study scanned at 0.50 μm/pixel (20×) were excluded in order to ensure availability of 40x for DLS-based MC and NP grading.

All primary invasive breast carcinoma cases with available slides or blocks were reviewed for inclusion. For TTH this includes all available cases from 2005 to 2016, for MLAB this includes cases from 2002 to 2011, and for TCGA data comprises the TCGA-BRCA study with cases from 1988 to 2013. The study was approved by the Advarra institutional review board (Columbia, Maryland) and deemed exempt from informed consent as all data were retrospective and de-identified.

### Whole slide image inclusion criteria

All available WSIs were reviewed by pathologists for slide-level inclusion criteria and quality assurance. Only slides containing H&E stained primary invasive breast carcinoma from formalin-fixed paraffin-embedded resection specimens were included in this study. Examples of excluded images include lymph node specimens, needle core biopsies, frozen tissue, immunohistochemistry slides, and slides containing carcinoma in-situ only.

This resulted in 1502 slides (657 cases) from TTH, 98 slides (98 cases) from MLAB, and 878 slides (829 cases) from TCGA. The slides from TTH were used for training and tuning of the models, slides from MLAB were used for tuning only, and TCGA slides represent a held-out external test set used only for evaluation of all models. For evaluating the individual DLS for each component feature, slides without a pathologist majority were excluded to ensure reliability of the reference for this performance evaluation, resulting in 685 slides / 662 cases. See Table 1 and Supplementary Fig. 1 for details regarding dataset usage and characteristics.

### Annotations

Pathologist annotations were performed for segmentation of invasive carcinoma as well as for all three components of the Nottingham histologic grading system (MC, NP, and TF). Annotations for the grading components were collected as slide-level labels as well as region-level labels for specific regions of tumor (three 1 mm × 1 mm regions per slide selected by pathologists to capture representative tumor). For both slide-level and region-level annotation tasks, 3 board-certified pathologists from a cohort of 10 pathologists were randomly assigned per slide, thus resulting in triplicate annotations per region of interest and per slide. More detail as follows.

#### Invasive carcinoma segmentation

All regions considered to represent invasive carcinoma were annotated, with guidance to provide coarse annotations capturing regions of at least 70% tumor purity (to account for non-tumor cells present in a given region). Regions considered to be carcinoma in-situ were also annotated and used to train the invasive carcinoma model. Slides determined on initial review to contain only carcinoma in situ, microinvasive tumor, lymphovascular invasion were excluded prior to invasive carcinoma annotation.

#### Mitotic figures

For each slide, the initial annotating pathologist selected three separate 1 × 1 mm^2^ regions with enriched mitotic count if present. Within each selected region, pathologists were asked to exhaustively annotate all mitotic figures. Each selected region was annotated by three pathologists, and only mitoses with agreement between at least two pathologists were used as “positive” events for training and evaluation.

#### Nuclear pleomorphism and tubule formation

For nuclear pleomorphism and tubule formation grading, we utilized the region boxes already selected during the mitotic figure annotation. Pathologists were asked to provide a component grade score for each selected region according to the Nottingham grading scale as though each region were independently representative of the whole tumor (acknowledging that such grading in clinical practice involves more holistic interpretation of tumor regions). Again, each region was annotated by three pathologists, and the final label for each region was based on the majority vote score.

#### Slide-level grading

Pathologists were asked to assess the whole slide on each component of the Nottingham grading scale, providing a 1–3 score for each component for each slide. Each slide was reviewed by three pathologists, and the majority vote was used to determine the slide-level label for training and evaluation. Additionally, as a separate source of grading, available pathology reports were reviewed and component grade scores from the original reports were recorded (referred to as “historic pathologist scores”).

### Deep learning system development

We developed 4 separate deep learning models: one to segment invasive carcinoma within a WSI, and three “grading models” to predict the slide-level component score for each of the three tumor features comprising the Nottingham combined histologic grade: MC, NP, and TF. The invasive carcinoma model was used to provide tumor masks for the Nottingham grading model.

The invasive carcinoma model was trained using pathologist annotations to distinguish between 3 classes: non-tumor, carcinoma in situ, and invasive carcinoma. The resulting model was evaluated on the tune set, achieving an AUC for invasive carcinoma vs. the other 2 classes (carcinoma in situ or non-tumor) of 0.95.

To segment invasive carcinoma versus the rest of tissue when applying DLS for histologic grading, we applied the argmax function to each patch (1024 by 1024 pixels) in the output likelihood map over the entire slides (with argmax referring to the class with the highest prediction score for each patch). Then, the patches for which the model estimated invasive carcinoma as the most likely class are selected as the invasive carcinoma segmentation for other downstream computing tasks (for e.g., slide-level MC, NP, and TF scores prediction).

For providing slide-level component scores, each model is used as part of a DLS that consists of two stages. The first stage (“patch-level”) tiles the invasive carcinoma mask regions of the WSI into individual patches for input into a convolutional neural network, providing as output a continuous likelihood score (0–1) that each patch belongs to a given class. For mitotic figure detection, this score corresponds to the likelihood of the patch corresponding to a mitotic figure. For NP and TF, model output is a likelihood score for each of the three possible grade scores of 1–3. All stage 1 models were trained using the data summarized in Table 1 and Supplementary Table 7 and utilizing ResNet50x1 pre-trained on a large natural image set (“JFT”)^39^. Stain normalization and color perturbation^18^ were applied and the models were trained until convergence. Hyperparameter configurations including patch size and magnification were selected independently for each component model using a combination of Vizier^40^ and sample grid search. Hyperparameters and optimal configurations for each stage 1 model are summarized in Supplementary Table 8.

The second stage of each DLS assigns a slide-level feature score (1–3) for each feature (MC, NP, and TF). This is done by using the stage 1 model output to train a lightweight classifier for slide-level classification. For MC, the stage 1 output is used to calculate “mitotic density” values over the invasive carcinoma region, and the mitotic density values corresponding to the 5th, 25th, 50th, 75th, and 95th-percentiles for each slide are used as the input features for the stage 2 model. Details regarding the conversion of patch-level stage 1 output to mitotic figure detection and mitotic density are provided in the following section. For NP and TF, the stage 2 input feature set is the mean patch-level output (mean softmax value) for each possible score (1, 2, or 3) across the invasive carcinoma region. Based on tune set results, logistic regression was selected for the stage 2 classifier for MC. For NP and TF, performance of different stage 2 approaches were comparable, including logistic regression, ridge regression, and random forest. Ridge regression was selected, due to its simplicity and the ease of generating continuous component scores with this approach. All classifiers are regularized with their regularization strengths chosen via a fivefold cross-validation on the training set. For NP, additional experiments with a hand-engineered nuclear segmentation-based approach were also conducted^13,14^. This approach did not improve performance in our experiments, potentially due to the wide variability in staining and cellular appearance in high-grade cases.

#### Mitotic figure detection and counting from patch-level stage-1 output

For the MC model, the output (likelihood score of mitotic figure) across all patches was considered as a heatmap that is used for downstream analysis. We thresholded this output probability to achieve a positive detection map. The detection threshold was set to 0.915 based on the tuning set. Because the mitosis annotations were provided by pathologists using 16 μm × 16 μm bounding boxes (about the size of one cell), we expected the detection of the model to be about the same size. To get the list of location of detection, we applied morphological erosion with a square structuring element of size 16 μm × 16 μm. This operation on two overlapping 16 µm × 16 µm regions will result in two disconnected points, allowing us to distinguish two nearby mitoses. We then performed connected-component analysis on the eroded map, and took the centroid of each connected component as the location of mitoses. This list of mitoses locations allowed counting of mitotic figures and the use of a sliding window approach to calculate the mitotic density. Mitotic density was calculated for all tiles across the entire invasive carcinoma mask (1.8 × 1.8 mm tiles with 50% overlapping stride). For evaluation, a predicted mitotic figure was considered a true positive event if there was a reference standard mitotic figure within 16 µm.

### Deep learning system evaluation

We evaluated DLS performance for histologic grading at both the patch level and the slide-level using the TCGA test set and the annotations described above. Patch-level evaluation corresponds to the stage 1 model output and utilizes the annotated regions of interest as the reference standard (three 1 mm × 1 mm regions per slide each annotated by three pathologists; see Annotations section of Methods above and Supplementary Methods). For MC, the patch-level reference standard corresponds to cell-sized regions identified by at least two of three pathologists as a mitotic figure. All other cell-sized regions not meeting these criteria were considered negative for the purposes of MC evaluation. For NP and TF, the majority vote annotation for each region of interest was assigned to all patches within that region and used as the reference standard (consistent with the approach for stage 1 training labels). The maximum probability in the model output probability map is selected to obtain the final per-patch prediction. For slide-level evaluation, the majority vote for each slide-level component score was used.

For patch-level evaluation, F1 score was calculated for mitosis detection and quadratic-weighted kappa was calculated for NP and TF. For slide-level evaluation, quadratic-weighted kappa was used for all components, including inter-pathologist agreement. Use of quadratic-weighted kappa was based on informative penalization of increased distance from the reference standard. Because the weighting scheme used in the literature is variable, additional kappa weighting schemes were also assessed and reported in Supplemental Table 1.

### Progression-free interval analysis

To further evaluate the histologic grading models, we also analyzed the prognostic value of DLS-based grading for predicting progression-free interval (PFI). PFI was chosen as a clinically meaningful endpoint suitable for TCGA-BRCA specifically as described previously^28^. Of note, 18 cases with PFI events in this data correspond to “new primary tumors”, predominantly of non-breast origin according to TCGA annotations. As such, and because disease-specific progression events following a new primary tumor could not be reliably identified from the available data, these cases were censored at the disease-free interval time in our analysis, resulting in the 829 cases and 93 events summarized in Table 1.

In planned analysis, we evaluated the prognostic value of the DLS and pathologist-provided scores both in isolation and in the context of available clinicopathologic features. As a pre-specified non-inferiority test (based on development set results), we compared the prognostic value of the sum of continuous DLS-based component scores versus the sum of discrete component scores provided by pathologists for the same images. Here, continuous scores refer to the model output corresponding to any fractional value between 1 and 3 for each component and discrete scores refer to the traditional integer scores of 1, 2, or 3. Combined histologic grade based on the summed scores (3–5: grade 1; 6–7; grade 2; 8–9 grade 3) was also evaluated in additional analyses. To conduct analyses adjusting for available baseline clinicopathologic variables (TNM, ER status, age) and calculate hazard ratios, we fit multivariable Cox regression models on the test set (TCGA-BRCA) using either the DLS-based component scores or the pathologist-provided component scores. To evaluate for improved prognostic value when adding AI-NGS information to the baseline variables, we performed likelihood-ratio tests for cox models fit on baseline clinicopathologic variables alone versus models fit on baseline variables combined with grading scores. The corresponding c-index for the risk scores provided by these models on the test were also calculated (as reported in Table 4).

In order to evaluate multivariable models for prognosis, we use leave-one-out cross-validation on the TCGA test set, fitting a Cox model per fold and calculating the mean C-index across folds. Because the absolute value of the risk score depends on many fitted parameters such as base hazard, which can vary, the risk scores may not be directly comparable across folds. To remedy this problem, the output risk score is normalized to a percentile with regard to the training data.

### Statistical analysis

Confidence intervals were generated via bootstrap resampling with 1000 samples. For patch-level and region-level evaluation of DLS, we performed bootstrap resampling over slides and for progression-free interval analysis, we performed bootstrap resampling over cases. All statistical tests are two-sided with the exception of the non-inferiority test, which is one-sided (with a pre-specified non-inferiority margin of 0.075 and alpha of 0.05). The margin was selected based on projected confidence intervals and power calculations using the tune dataset. No adjustment for multiple comparisons was implemented. For Ki-67 correlation analysis of mitotic count and Ki-67, permutation testing between DLS and Pathologists MC score was performed with 1,000 samples. C-indices were computed using the lifelines.utils.concordance_index function in the Python Lifelines package (v0.26.0) and additional analyses were performed using Python (v3.7.10), Numpy (v1.19.5), and scikit-learn (v0.24.1) libraries.

## Supplementary information

Supplementary Information

## Data Availability

TCGA data utilized in this study is publicly available via the Genomic Data Commons Data Portal (gdc.cancer.gov). The tertiary hospital data was used under a Defense Health Agency data sharing agreement. Requests regarding data can be directed to the Defense Health Agency Privacy Office at DHA.PrivacyOfficeMail@mail.mil. The medical laboratory dataset is not publicly available at this time due to data privacy considerations

## Code availability

The deep learning framework (TensorFlow v1.14) used in this study is available at https://github.com/tensorflow/tensorflow/tree/r1.14. The convolutional neural network ResNet50x1 is available at https://github.com/google-research/big_transfer/blob/master/bit_tf2/models.py and instructions for downloading pretrained models can be found at https://github.com/google-research/big_transfer. The stage 2 models were implemented with scikit-learn (v0.24.0), available at https://github.com/scikit-learn/scikit-learn/tree/0.24.X. Details and code for stage 2 featurization and utlization is available at https://github.com/Google-Health/google-health/tree/master/breast_survival_prediction. The final, trained models have not yet undergone regulatory review and cannot be made available at this time. Interested researchers can contact P.-H.C.C. for questions on its status and access.
