# Supplementary material for: Deep learning models for histologic grading of breast cancer and association with disease prognosis
Source: NPJ Breast Cancer. 2022 Oct 4;8:113. doi: 10.1038/s41523-022-00478-y (PMC9530224; doi:10.1038/s41523-022-00478-y)
Supplement: Supplementary file 1 — Supplementary Information [file 41523_2022_478_MOESM1_ESM.pdf]

Supplementary Information

Deep learning models for histologic grading of breast cancer and association with disease prognosis

Authors

Ronnachai Jaroensri[1], Ellery Wulczyn[1], Narayan Hegde[1], Trissia Brown[2], Isabelle Flament-Auvigne[2], Fraser Tan[1], Yuannan Cai[1], Kunal Nagpal[3], Emad A. Rakha[4], David J. Dabbs[5], Niels Olson[6], James H. Wren[7], Elaine E. Thompson[7], Erik Seetao[7], Carrie Robinson[8], Melissa Miao[9], Fabien Beckers[9], Greg S. Corrado[1], Lily H. Peng[1], Craig H. Mermel[1], Yun Liu[1], David F. Steiner[1,†,*], Po-Hsuan Cameron Chen[1,†,*]

[1]Google Health, Palo Alto, CA, USA
[2]Work done at Google Health via Vituity, Emeryville, CA, USA
[3]Work done at Google Health, current affiliation Tempus Labs Inc, Chicago, Illinois, United States of America
[4]Department of Pathology, School of Medicine, University of Nottingham, Nottingham, UK
[5]John A. Burns University of Hawaii Cancer Center, Honolulu, HI, USA, and Department of Pathology, Magee-Womens Hospital of UPMC, Pittsburgh, PA, USA
[6]Defense Innovation Unit, Mountain View, CA, USA and Uniformed Services University, Bethesda, MD, USA
[7]Henry M. Jackson Foundation, Bethesda, MD, USA
[8]Laboratory Department, Naval Medical Center San Diego, San Diego, CA, USA
[9]Verily Life Sciences, South San Francisco, CA, USA

[†]Equal contribution
[*]Corresponding authors

Supplementary Tables

Supplementary Table 1. Additional metrics for performance of component grading models (associated with Table 2-3)

Component models	Metric	Result [95% CI]
Mitotic Count (patch-level)	Precision	0.76 [0.74, 0.78]
	Recall	0.50 [0.47, 0.52]
Nuclear Pleomorphism (patch-level)	Linear-weighted Kappa	0.42 [0.38, 0.46]
	Unweighted Kappa	0.40 [0.35, 0.43]
	Accuracy	0.67 [0.64, 0.69]
	Balanced Accuracy	0.50 [0.48, 0.52]
Tubule Formation (patch-level)	Linear-weighted Kappa	0.61 [0.55, 0.67]
	Unweighted Kappa	0.54 [0.47, 0.59]
	Accuracy	0.93 [0.91, 0.94]
	Balanced Accuracy	0.67 [0.61, 0.72]
Mitotic Count (slide-level)	Linear-weighted Kappa	0.73 [0.69, 0.77]
	Unweighted Kappa	0.63 [0.58, 0.67]
Nuclear Pleomorphism (slide-level)	Linear-weighted Kappa	0.40 [0.35, 0.45]
	Unweighted Kappa	0.36 [0.30, 0.41]
Tubule Formation (slide-level)	Linear-weighted Kappa	0.69 [0.62, 0.75]
	Unweighted Kappa	0.64 [0.57, 0.71]

Supplementary Table 2A: Slide-level benchmarks for grading agreement in breast cancer.

Source	Kappa metric weighting	Histologic Feature		
		MC	NP	TF
Inter-pathologist agreement				
Rakha et al., 2017[2]	Unweighted	0.34	0.36	0.49
Robbins et al., 1995[3]	Unweighted	0.69	0.46	0.52
Frierson et al., 1995[4]	Unweighted	0.52	0.40	0.64
Mercan et al., 2020[5]	Quadratic	N/A	0.35-0.49	N/A
Ginter et al., 2021[6]	Fleiss	0.28	N/A	0.50
Current Study (inter-pathologist average)	Quadratic	0.56	0.36	0.55
Current Study (DLS-pathologist average)	Quadratic	0.64	0.39	0.69
Current Study (DLS-majority vote path)	Quadratic	0.81	0.48	0.75
Algorithm-reference standard agreement				
Veta et al., 2019 (Original TUPAC 16 publication)[7]	Fleiss	0.567	N/A	N/A
Mercan et al., 2020[5]	Quadratic	N/A	0.43-0.56	N/A

Supplementary Table 2B: Comparison of selected studies that perform survival analysis.

Source	Summary
Wang et al.[8]	Weakly supervised learning for whole slide images to classify Nottingham grade 1 and 3. Specific focus on prognostic stratification of Nottingham grade 2 cases. Area under the curve analysis for Grade 1 vs. Grade 3 classification. Kaplan-Meir and hazard ratio analysis on Grade 2 cases an external test set
Lu et al. [1]	Prognostic evaluation of computationally extracted morphologic nuclear features using a tissue microarray (TMA) cohort of 276 ER+ lymph node negative cases
Rakha et al.[9]	Human pathologist grading on a cohort of approximately 2000 cases from the University of Nottingham is shown to be strongly associated with breast cancer-specific survival and disease-free survival by Kaplan-Meir analysis and log-rank tests
Present work	AI-based grading algorithm involving the three Nottingham grading system components. C-index analysis (c-index=0.58) for progression-free interval with non-inferiority testing to pathologist-based grading using the TCGA-BRCA cohort as an external validation set.

Supplementary Table 3: Tune set prognostic performance for direct risk prediction using histologic scoring provided by DLS and pathologists

Scoring method	C-index (All cases; n=348)	
	DLS	Pathologist
Summed Score Continuous [3.00 - 9.00]	0.62 [0.54, 0.69]	N/A
Summed Score Discrete [3,4,5,6,7,8,9]	0.62 [0.54, 0.69]	0.59 [0.51, 0.66]
Combined Histologic Grade [1,2,3]	0.61 [0.54, 0.68]	0.57 [0.50, 0.63]

These tuning set data were used to select configuration for planned primary analysis

Supplementary Table 4: C-index for histologic score using alternate configurations of DLS and pathologist scoring (direct risk prediction without incorporating clinical variables)

Supplementary Table 4A: All Cases

**Histologic grade score configuration**	**Values**	**C-index (All cases; n=829) [95% CI]**		**C-index (ER+ only; n=593) [95% CI]**		**C-index (ER+/HER2-; n=479) [95% CI]**	
		DLS	Pathologist	DLS	Pathologist	DLS	Pathologist
Summed Score Discrete	Integer in [3,9]	0.59 [0.53, 0.64]	0.58 [0.51, 0.63]*	0.61 [0.55, 0.68]	0.58 [0.50, 0.65]	0.62 [0.55, 0.70]	0.57 [0.47, 0.66]
Combined Histologic Grade	[1,2,3]	0.60 [0.55, 0.65]	0.58 [0.51, 0.63]	0.62 [0.56, 0.69]	0.58 [0.50, 0.65]	0.63 [0.56, 0.70]	0.57 [0.48, 0.66]
Summed Score Continuous	Float Number in [3,9]	0.58 [0.52, 0.64]*	N/A	0.58 [0.51, 0.66]	N/A	0.57 [0.48, 0.65]	N/A
Summed Score Average	0.333 increments [3,9]	N/A	0.61 [0.55, 0.66]	N/A	0.62 [0.54, 0.69]	N/A	0.63 [0.55, 0.71]
Majority-voted Summed Score	Integer in [3,9]	N/A	0.61 [0.54, 0.66]	N/A	0.63 [0.55, 0.70]	N/A	0.63 [0.55, 0.72]
Majority-voted Histologic Grade	[1,2,3]	N/A	0.60 [0.53, 0.65]	N/A	0.63 [0.56, 0.70]	N/A	0.62 [0.54, 0.74]

Supplementary Table 4B: Only cases with available pathology report scoring

**Histologic grade score configuration**	**Values**	**C-index (cases with path report only; n=550) [95% CI]**		**C-index (cases with path report *and* ER+ only; n=426) [95% CI]**		**C-index (cases with path report *and* ER+/HER2- only; n=342) [95% CI]**	
		DLS	Pathologist	DLS	Pathologist	DLS	Pathologist
Summed Score Discrete	Integer in [3,9]	0.60 [0.53, 0.67]	0.57 [0.49, 0.65]	0.63 [0.54, 0.72]	0.56 [0.46, 0.68]	0.64 [0.54, 0.75]	0.56 [0.42, 0.71]
Combined Histologic Grade	[1,2,3]	0.61 [0.54, 0.67]	0.57 [0.49, 0.64]	0.63 [0.55, 0.73]	0.56 [0.46, 0.66]	0.64 [0.54, 0.74]	0.56 [0.43, 0.69]
Summed Score Continuous	Float Number in [3,9]	0.58 [0.50, 0.66]	N/A	0.57 [0.47, 0.68]	N/A	0.57 [0.44, 0.69]	N/A
Summed Score Average	0.333 increments [3,9]	N/A	0.61 [0.53, 0.68]	NA	0.61 [0.51, 0.71]	NA	0.62 [0.50, 0.74]
Majority-voted Summed Score	Integer in [3,9]	N/A	0.61 [0.53, 0.69]	N/A	0.61 [0.51, 0.71]	N/A	0.62 [0.50, 0.74]
Majority-voted Histologic Grade	[1,2,3]	N/A	0.60 [0.52, 0.67]	N/A	0.62 [0.52, 0.71]	N/A	0.61 [0.49, 0.73]
Pathology Report Summed Score	Integer in [3,9]	N/A	0.62 [0.54, 0.70]	N/A	0.62 [0.50, 0.73]	N/A	0.62 [0.49, 0.74]
Pathology Report Histologic Grade	[1,2,3]	N/A	0.60 [0.52, 0.68]	N/A	0.60 [0.49, 0.71]	N/A	0.62 [0.50, 0.74]

To avoid comparing performance on different cases when evaluating original pathology report data, these data represent only the subset of cases for which original pathology reports were available (n=550 for all cases, and n=426 for ER+ only,)

Supplementary Table 5. Prognostic performance for using individual components of histologic grade

	**c-index (full dataset; n=829)**	**c-index (ER+; n=593)**	**c-index (ER+/HER2-; n=479)**
**DLS**			
Continuous MC	0.59 [0.53, 0.65]	0.60 [0.52, 0.69]	0.60 [0.49, 0.70]
Continuous NP	0.55 [0.48, 0.61]	0.54 [0.46, 0.62]	0.51 [0.43, 0.60]
Continuous TF	0.49 [0.43, 0.55]	0.48 [0.40, 0.55]	0.48 [0.41, 0.56]
Discrete MC	0.58 [0.53, 0.64]	0.60 [0.53, 0.68]	0.62 [0.53, 0.70]
Discrete NP	0.52 [0.47, 0.57]	0.51 [0.46, 0.58]	0.51 [0.45, 0.57]
Discrete TF	0.54 [0.50, 0.57]	0.54 [0.49, 0.59]	0.54 [0.48, 0.60]
**Pathologist**			
Single Pathologist MC	0.54 [0.48, 0.59]	0.52 [0.44, 0.60]	0.52 [0.43, 0.62]
Single Pathologist NP	0.56 [0.51, 0.61]	0.57 [0.49, 0.64]	0.54 [0.46, 0.62]
Single Pathologist TF	0.55 [0.51, 0.58]	0.56 [0.51, 0.60]	0.56 [0.50, 0.62]
Majority voted MC	0.58 [0.52, 0.63]	0.59 [0.52, 0.67]	0.61 [0.52, 0.69]
Majority voted NP	0.57 [0.52, 0.63]	0.59 [0.52, 0.66]	0.57 [0.50, 0.65]
Majority voted TF	0.55 [0.52, 0.58]	0.55 [0.50, 0.59]	0.56 [0.50, 0.61]
Average MC	0.58 [0.52, 0.64]	0.60 [0.52, 0.68]	0.62 [0.53, 0.70]
Average NP	0.58 [0.52, 0.64]	0.57 [0.48, 0.65]	0.56 [0.46, 0.65]
Average TF	0.56 [0.52, 0.61]	0.57 [0.50, 0.63]	0.57 [0.50, 0.64]
Historical MC	0.65 [0.57, 0.71]	0.65 [0.54, 0.74]	0.67 [0.56, 0.78]
Historical NP	0.55 [0.47, 0.61]	0.54 [0.44, 0.64]	0.52 [0.41, 0.62]
Historical TF	0.52 [0.46, 0.57]	0.51 [0.43, 0.58]	0.50 [0.41, 0.60]

MC: Mitotic Count; NP: Nuclear Pleomorphism; TF: Tubule Formation

Supplementary Table 6: Prognostic performance using combination of histologic components and baseline clinical and individual component features.

Model features	c-index	p-value (Likelihood Ratio Test) for adding features to baseline
Baseline Features Only	0.74 [0.67, 0.81]	N/A (reference)
**Mitotic Count Score**		
Baseline + AI-NGS MC	0.75 [0.69, 0.81]	0.041
Baseline + Single Pathologist MC	0.74 [0.68, 0.81]	0.551
Baseline + Majority Pathologist MC	0.75 [0.68, 0.81]	0.246
**Nuclear Pleomorphism Score**		
Baseline + AI-NGS NP	0.74 [0.68, 0.81]	0.687
Baseline + Single Pathologist NP	0.75 [0.69, 0.81]	0.125
Baseline + Majority Pathologist NP	0.75 [0.69, 0.81]	0.018
**Tubule Formation Score**		
Baseline + AI-NGS TF	0.75 [0.69, 0.81]	0.053
Baseline + Single Pathologist TF	0.75 [0.68, 0.81]	0.015
Baseline + Majority Pathologist TF	0.75 [0.69, 0.81]	0.070

Cox models were fit and evaluated directly on the test set and p-values are for likelihood ratio test of baseline versus baseline plus grading scores. Baseline features include age (continuous), TNM (categorical), and ER status (binary). Number of cases represents all cases with baseline characteristics available (n=762 cases; 82 events). Confidence intervals computed via bootstrap with 1000 iterations.

Supplementary Table 7: Annotation Summary

		Train/Tune	Test
Mitotic Count	Mitoses Annotated	3,641	9,678
	Area reviewed	1,979 mm^2	2,193 mm^2
Nuclear Pleomorphism	Grade 1	207k patches/ 53 mm^2	323k patches/ 83 mm^2
	Grade 2	3.91M patches/ 1,001 mm^2	4.37M patches/ 1,119 mm^2
	Grade 3	2.30M patches/ 589 mm^2	3.44M patches/ 881 mm^2
Tubule Formation	Grade 1	380k patches/ 97 mm^2	226k patches/ 58 mm^2
	Grade 2	1.04M patches/ 265 mm^2	719k patches/ 184 mm^2
	Grade 3	4.94M patches/ 1,264 mm^2	6.97M patches/ 1,785 mm^2

Supplementary Table 8: Hyperparameters used for model training.

Hyperparameters		Invasive Carcinoma	Mitotic Count	Nuclear Pleomorphism	Tubule Formation
Network configuration	Architecture	BiT-L (ResNet 50x1)			
	Loss function	softmax cross-entropy			
Model Inputs	Magnification	10x	40x	40x	10x
	Patch size	1024	128	1024	1024
	Stain normalization	Stain normalization applied using reference color statistics from a fixed slide.			
Data augmentation	Orientation	Left/right mirroring and all 4 rotations			
	Brightness	tf.image.random_brightness with max_delta=0.25			
	Saturation	tf.image.random_saturation with lower=0.75 and upper=1.25			
	Hue	tf.image.random_hue with max_delta=0.04			
	Contrast	tf.image.random_contrast with lower=0.25 and upper=1.75			
Training	Batch size	32	32	8	32
	Training steps	1M with early stopping			
Optimizer configuration	Optimizer	RMSProp			
	Decay	0.97	0.95	0.95	0.97
	Epsilon	0.001	0.001	0.001	0.001
	Momentum	0.7	0.9	0.7	0.7
Learning rate	Initial Rates	3e-5	1e-5	3e-6	3e-4
	Exp. decay	0.97	0.95	0.9	0.95
	Decay steps	2,000	20,000	2,000	10,000

Supplementary Figures

Supplementary Figure 1

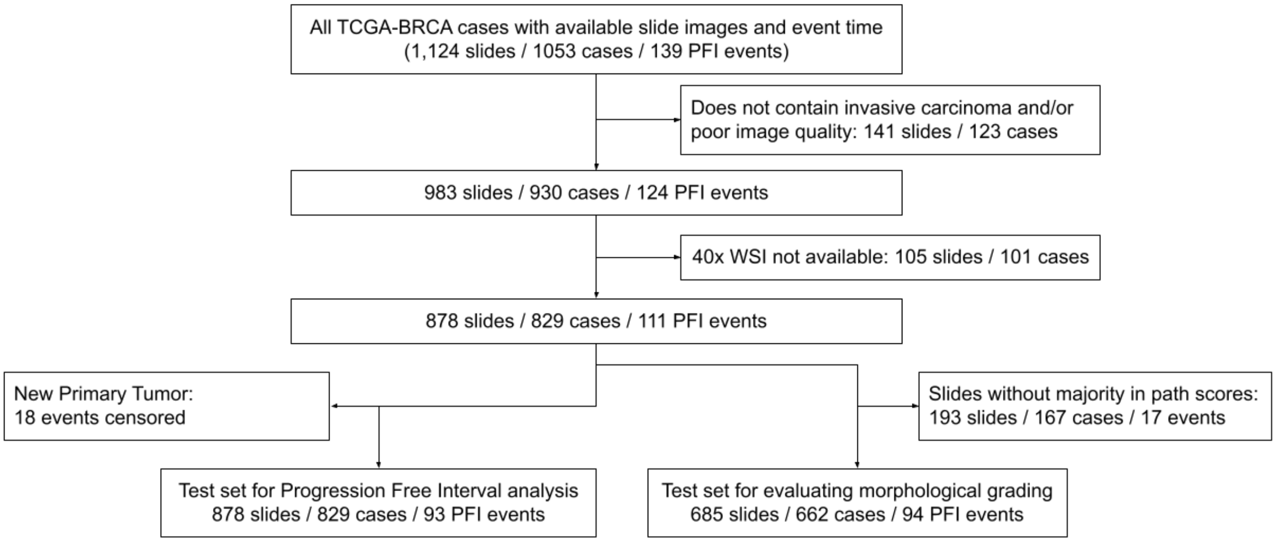

Supplementary Figure 1: STARD Diagram for test dataset (TCGA-BRCA). Diagram depicts TCGA-BRCA test sets used for prognostic analysis (Progression Free Interval Analysis Test Set) and evaluation of performance for individual grading algorithms (Nottingham Score Test Set).

Supplementary Figure 2

A

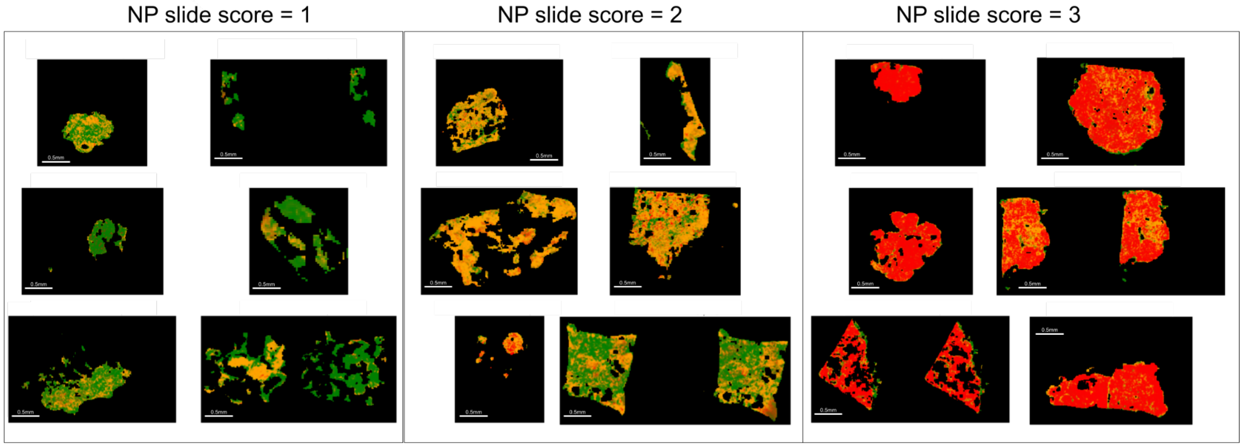

Patch-level legend:
Green: Score = 1
Orange: Score = 2
Red: Score = 3

B

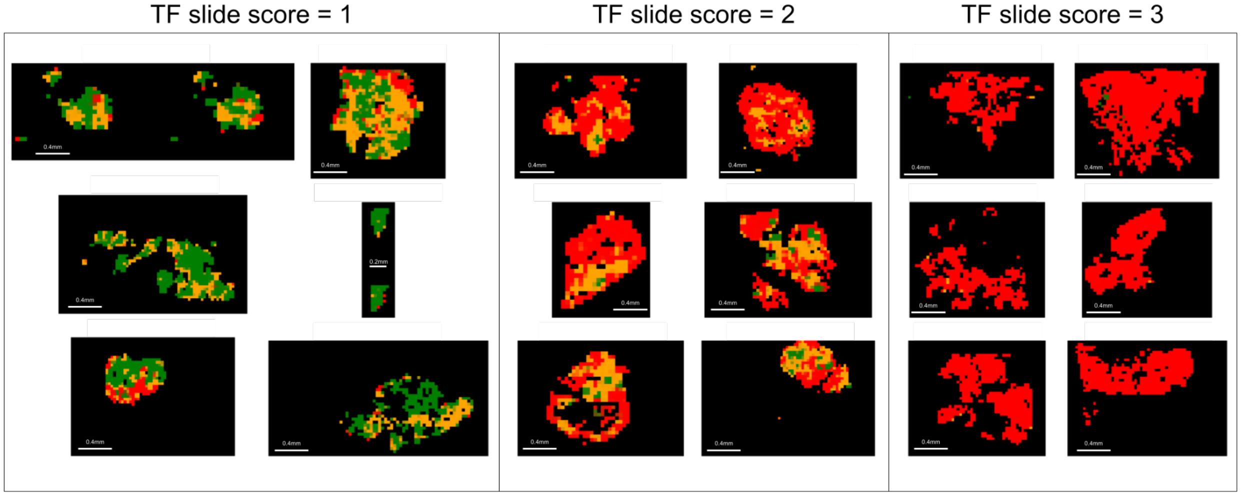

Patch-level legend:
Green: Score = 1
Orange: Score = 2
Red: Score = 3

Supplementary Figure 2: Patch-level classification of nuclear pleomorphism and tubule formation across whole slide images. Patch-level predictions for nuclear pleomorphism (A) and tubule formation (B) across whole slide images are shown. These are randomly sampled slides for which the slide-level score matched the majority vote pathologist slide-level score. Only regions of invasive carcinoma as identified by the invasive carcinoma model are shown. Green represents individual patches classified (argmax) with score of 1, yellow with score of 2, and red with score of 3. Patch size is 256 μm x 256 μm for nuclear pleomorphism model and 1 mm x 1 mm for tubule formation model.

Supplementary Figure 3

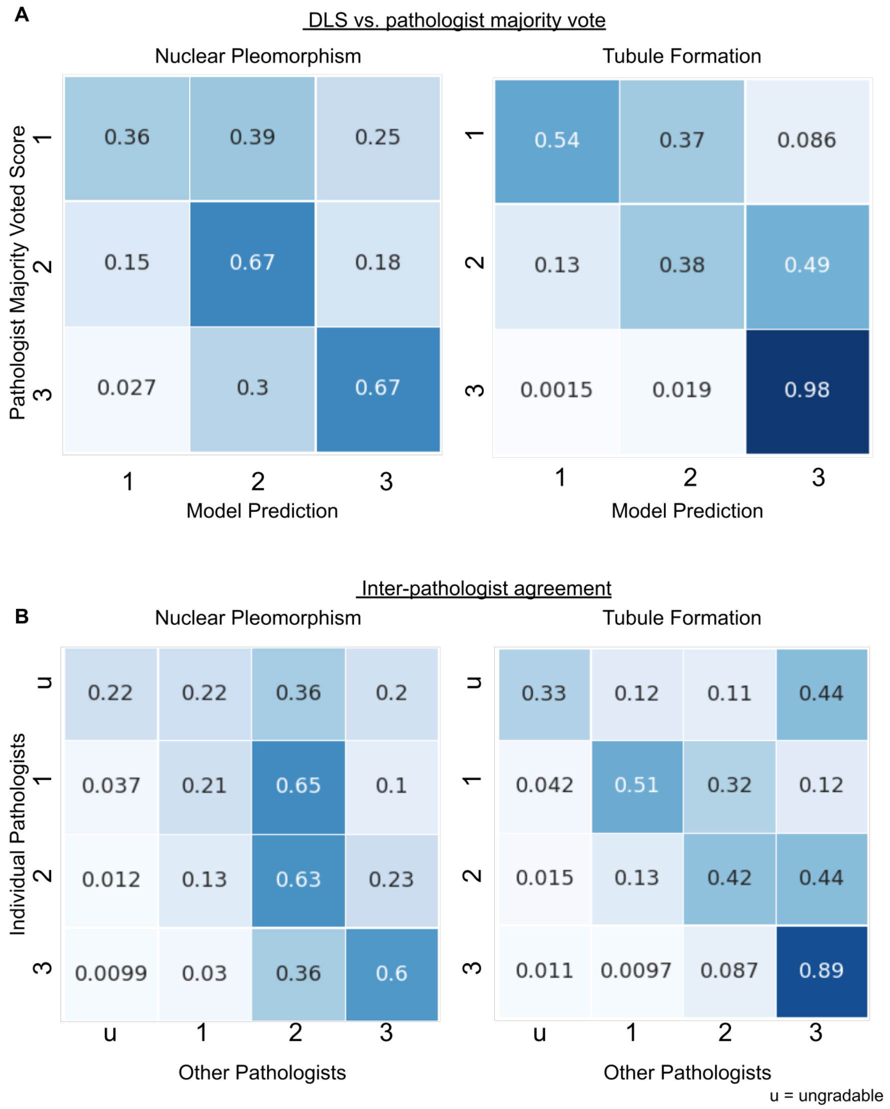

Supplementary Figure 3: Region-level DLS and inter-pathologist agreement. DLS-pathologist agreement (A) and interpathologist agreement (B) for specific regions of tumor. In panel A the deep learning system (DLS) output (columns) is compared to the Pathologist majority vote (rows). In panel B, the pathologist scores themselves contribute to the majority vote and thus a direct comparison to panel A cannot be made. As such, we calculate the confusion matrices between each individual pathologist (rows), and the rest of pathologists that grade the same regions (columns). Then we take the average of these confusion matrices across all pathologists to arrive at the data shown. This was done to summarize the average agreement between each pathologist and the rest of the cohort.

Supplementary Figure 4

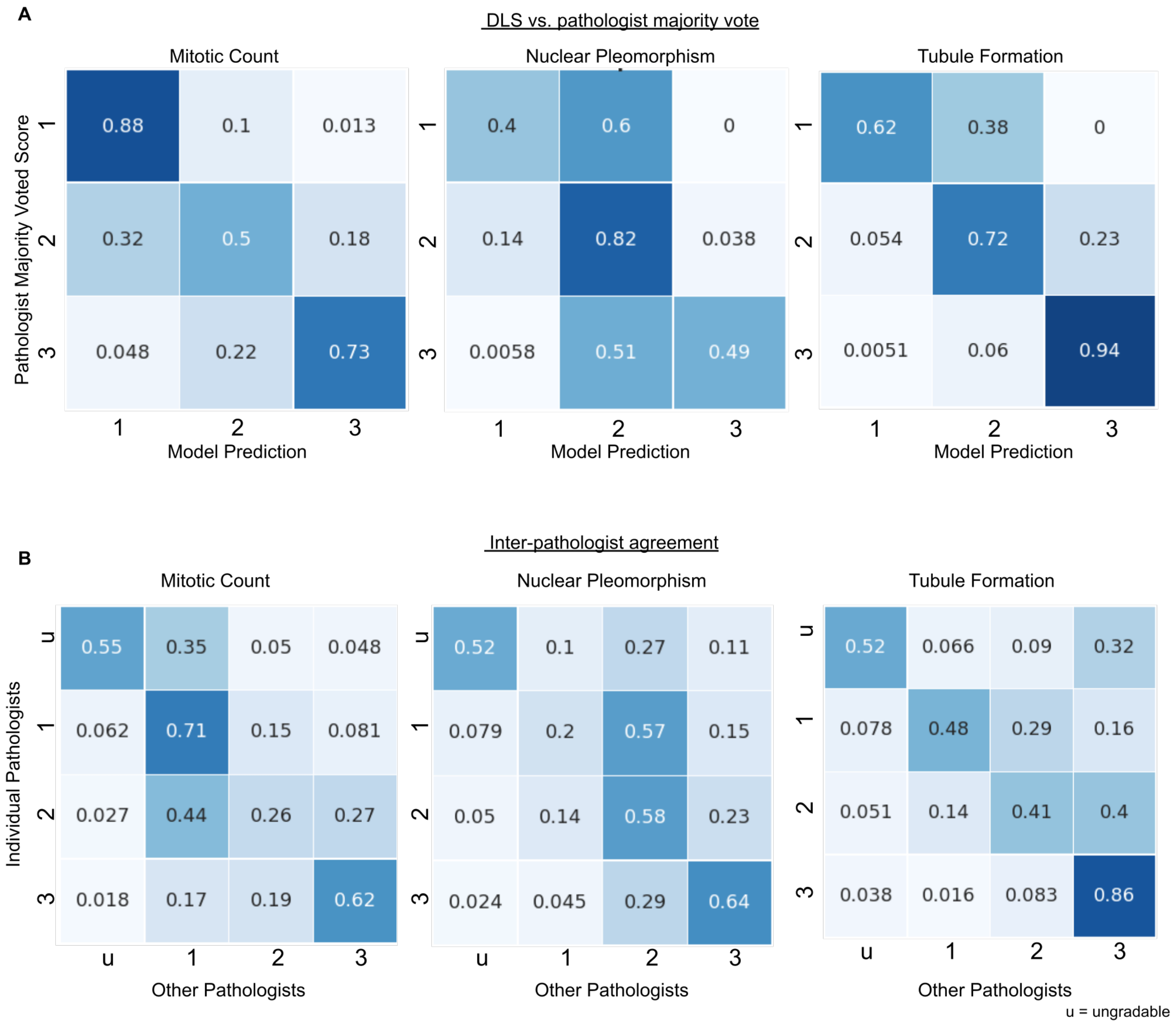

Supplementary Figure 4: Slide-level DLS and inter-pathologist agreement. DLS-pathologist agreement (A) and interpathologist agreement (B) for individual whole slide images. In panel B, we first calculate the confusion matrices between each individual pathologist (rows), and the rest of pathologists that grade the same regions (columns). Then we take the average of these confusion matrices across all pathologists to arrive at the data shown. This was done to summarize the average agreement between each pathologist and the rest of the cohort.

Supplementary Figure 5

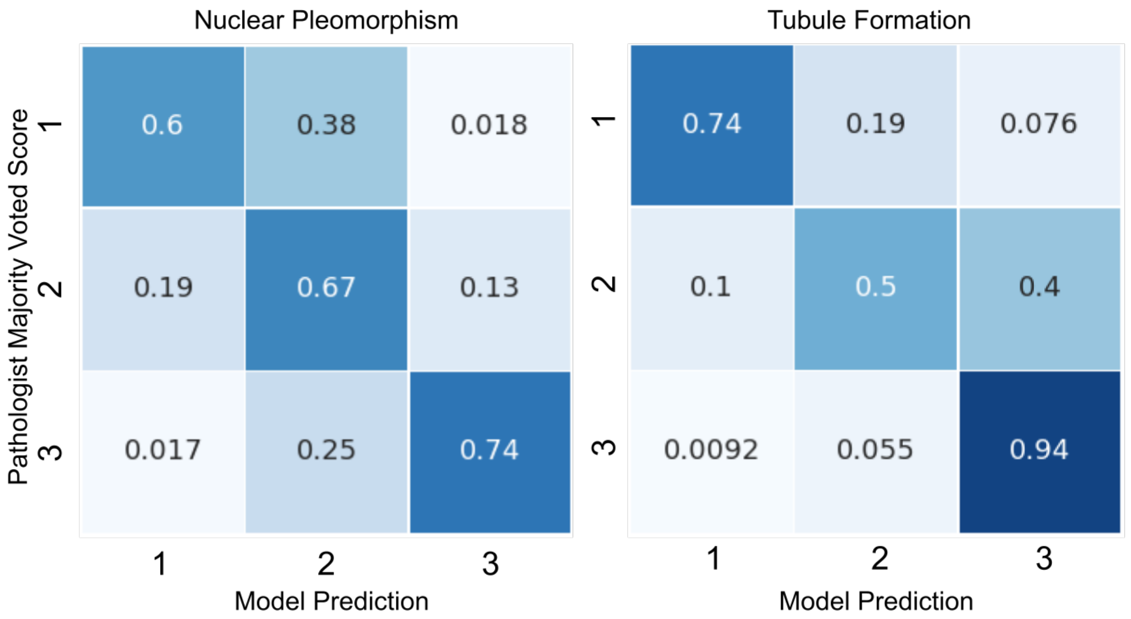

Supplementary Figure 5: Patch level DLS and pathologist agreement for the tune set. Model agreement with the majority vote score for individual regions are shown for nuclear pleomorphism and tubule formation, respectively. Values represent a portion of cases for each reference score with the corresponding model score.
